# Short- and Long-Term Repeated Forced Swim Stress Induce Depressive-Like Phenotype in Mice: Effectiveness of 3-[(4-Chlorophenyl)Selanyl]-1-Methyl-1H-Indole

**DOI:** 10.3389/fnbeh.2020.00140

**Published:** 2020-08-27

**Authors:** Ana Paula Pesarico, Paloma T. Birmann, Rodrigo Pinto, Nathalia Batista Padilha, Eder João Lenardão, Lucielli Savegnago

**Affiliations:** ^1^Research Group on Neurobiotechnology—GPN, CDTec, Federal University of Pelotas, UFPel, Pelotas, Brazil; ^2^Laboratory of Clean Organic Synthesis—LASOL, CCQFA, Federal University of Pelotas, Pelotas, Brazil

**Keywords:** selenium, repeated forced swim stress, depression, oxidative stress, corticosterone

## Abstract

Exposure to stress highly correlates with the emergence of mood-related illnesses. Therefore, the present study was designed to characterize the acute and chronic effects of 3-((4-chlorophenyl)selanyl)-1-methyl-1H-indole (CMI) on depressive-like behavior induced by repeated forced swim stress (FSS) in male adult *Swiss* mice. In the repeated FSS, mice were placed in water to swim for a single trial during a 15-min period. Twenty-four hours after the first FSS, the animals were placed in water to swim through a series of four trials, and each of them swam for 6 min long; between each trial, mice were towel dried and returned to their home cage for 6 min. In addition, the oxidative stress in the prefrontal cortex and hippocampus and corticosterone levels of plasma of mice were investigated. The animals exposed to FSS were treated with CM in two different protocols. In protocol 1, CMI [1 and 10 mg/kg, intragastric (i.g.) route] or fluoxetine, a positive control (10 mg/kg, i.g. route), were administered 30 min before of sections of repeated FSS in both days of stress. After the last section of repeated FSS, the mice performed first the spontaneous locomotor activity and after the tail suspension test. In protocol 2, CMI or fluoxetine (1 mg/kg, i.g. route) was administered for 20 days after the exposition of repeated FSS. The spontaneous locomotor activity, tail suspension, and forced swimming tests were performed in this order after 24 h of last administration of CMI or fluoxetine. The euthanasia of animals was performed after the behavioral tests. CMI and fluoxetine abolished the depressive-like behavior induced by repeated FSS in mice in the two different treatments. CMI modulated the oxidative stress in the prefrontal cortices and hippocampi of mice subjected to repeated FSS. Mice subjected to repeated FSS had an increase in the corticosterone levels and CMI regulated the levels of this glucocorticoid. These findings demonstrate that CMI was effective to abolish the depressive-like behavior induced by repeated FSS, which was accompanied by changes in the corticosterone levels and oxidative stress of prefrontal cortices and hippocampi of mice.

## Introduction

The synthetic organoselenium compound 3-((4-chlorophenyl)selanyl)-1-methyl-1H-indole (CMI) has been well studied by our research group. The first study demonstrated that CMI presents antioxidant activity (Vieira et al., 2017). Subsequent studies showed its antidepressant-like propriety in the inflammatory model with the involvement of oxidative stress and reduction of neuroinflammation (Casaril et al., 2017) and its antinociceptive effect mediated by the monoaminergic, opioidergic, and adenosinergic systems (Birmann et al., 2018). In the previous results, the serotonergic system was suggested as a mechanism of the effect of CMI in the depression—anxiety comorbidity (Casaril et al., 2019b). More recently, CMI reversed behavioral and biochemical alterations in the dyad pain—depression induced by partial sciatic nerve ligation and possibly modulation of the oxidative system (Birmann et al., 2019), and CMI treatment also attenuates depression and cognitive impairment in 4T1 tumor-bearing mice (Maria Casaril et al., 2019). All these results instigated us to research more about the CMI treatment in stress models.

Several preclinical models have been developed to assess the effects of stress on depression-related behaviors (Duman and Monteggia, 2006; Zhang et al., 2015; Wang et al., 2017) because environmental stressors, such as interpersonal conflict and trauma, have been recognized to contribute to the development of mental disorders, including depression. Interestingly, most experimental paradigms study the effects acute of stress. However, a growing body of literature suggests that the long-term effects of stressors can damage health and may also play a critical role in the etiology of depression (Schneiderman et al., 2005).

The mechanisms that mediate this association, stress and depressive disorder, have yet to be determined, but there is growing evidence suggesting that a constellation of physiological responses, including nervous and endocrine systems, are the key mechanisms through which stress leads to the development of depressive symptoms (Tsigos et al., 2000). Among those responses, the oxidative stress and hyperactivity of hypothalamic-pituitary-adrenal (HPA) axis are two of the most common neurobiological changes in depressive patients (Holsboer, 2000; Michel et al., 2012).

Oxidative stress consists in the excess of reactive species (RS) and situations where antioxidant defenses are compromised. RS may react with macromolecules of the cell like fatty acid, DNA, and protein, causing damage to these macromolecules. Furthermore, the brain, owing to its high metabolic rate, is one of the most vulnerable organs to the damaging effects of RS. This may explain RS involvement in several neuropsychiatric diseases (Maes et al., 2009, 2011; Salim, 2017). The prefrontal cortex and hippocampus, specifically, are associated to the regulation of emotion and responses to stress (Drevets et al., 2008).

Given the aforementioned considerations, the focus of this study was: (1) to design a model of long-term stress using repeated forced swim stress (FSS); and (2) to investigate the effect of CMI on the depressive-like behavior induced by repeated FSS in mice, either soon after stress or long-lasting stress. In addition, it was investigated whether the CMI effects on depressive-like behavior are accompanied by modulation of oxidative stress in the prefrontal cortex and hippocampus and on the corticosterone levels in the plasma of stressed mice.

## Materials and Methods

### Animals

The experiments were carried out using male adult *Swiss* mice (25–30 g, 2 months old) from our breeding colony. The animals were housed in cages (five mice per cage) with free access to food and water. They were kept in a separate animal room with controlled temperature (22 ± 2°C) on a 12-h light/12-h dark cycle, and the lights were turned on every day at 7:00 a.m. Commercial diet (Guaiba, Brazil) and tap water were supplied *ad libitum*. The present experimental study was approved by the Institutional Ethics Committee on Care and Use of Experimental Animal Resources from the Federal University of Pelotas, Brazil and registered under the number 135/2018. The procedures in this study were performed in accordance with the NIH Guide for the Care and Use of Laboratory Animals. All efforts were made to minimize animal suffering and to reduce the number of animals used in the experiments.

### Drugs

**Figure 1:**
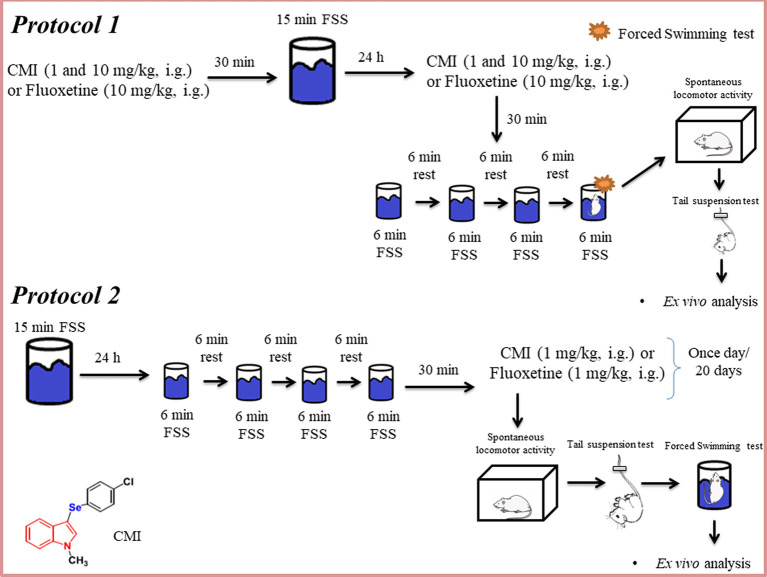
Schematic representation of the experimental design of this study. FSS, forced swim stress; CMI, 3-((4-chlorophenyl)selanyl)-1-methyl-1H-indole; i.g., intragastric.

CMI (Figure 1) was prepared and characterized at the Laboratory of Clean Organic Synthesis at the Federal University of Pelotas, according to Vieira et al. (2015).

Fluoxetine and all other chemicals were of analytical grade and were obtained from Servylab and WF Científica (Brazil). CMI was dissolved in canola oil (a non-polar and inert substance) and fluoxetine was dissolved in saline, and both compounds were administered by intragastric route at a constant volume of 10 ml/kg body weight.

In the present study, fluoxetine was used with the aim to validate the behavioral tests and it was not used with the intent to compare its effect with CMI. Possibly, the effect of CMI and fluoxetine cannot be compared directly because both are pharmacokinetically different. More studies are necessary to elucidate this hypothesis.

In protocol 1, the animals were administered 1 and 10 mg/kg of CMI, which was the dose found to have a better effect in repeated FSS. In protocol 2, considering that animals would receive 20 times the compound (20 days, once a time/day), our research group decided use the dose of 1 mg/kg CMI based on the previous study with organic compounds (Pesarico et al., 2016). Regarding the fluoxetine treatment, in the first protocol, we used 10 mg/kg of fluoxetine because previous studies demonstrated that acute administration of fluoxetine had no effect in doses lower than 10 mg/kg, whereas in protocol 2 we used 1 mg/kg because chronic administration of fluoxetine has a significant effect in the behavioral tests (Ratajczak et al., 2019; Gall et al., 2020).

### Repeated FSS

The repeated FSS (Rosa et al., 2018) was used as a repeated and inescapable stress, which results in a progressive increase in the amount of time the animal remains immobile. The mice were placed in water to swim for a single trial, during a 15-min period (day 1). Twenty-four hours after the first FSS, animals were placed in water to swim through a series of four trials, and each of them swam for 6 min long; between each trial, mice were towel dried and returned to their home cage for 6 min (day 2).

### Experimental Protocol

This study was divided into two protocols. All analyses were performed by an independent investigator blinded to the experimental conditions.

#### Protocol 1

The first protocol aimed to investigate the antidepressant-like effect of acute CMI in the repeated FSS model (short-term stress). Animals were randomly divided into seven experimental groups. CMI was administered at doses of 1 and 10 mg/kg and fluoxetine was administered at a dose of 10 mg/kg 30 min before the first FSS session (day 1; *n* = 7 animals/group, especially fluoxetine group has 6 animals/group). Twenty-four hours after (day 2), the mice received the same doses of CMI and the same dose of fluoxetine 30 min before the subsequent repeated FSS sessions (Figure 1). Fluoxetine was used with the objective of validating the stress model. The control group and repeated FSS group received two vehicles (saline and canola oil).

After the repeated FSS, the animals performed the behavioral tests. The forced swimming test (FST) of repeated FSS group, 1 mg/kg CMI + repeated FSS group, 10 mg/kg CMI + repeated FSS group, and 1 mg/kg fluoxetine + repeated FSS group was performed in the last fourth trial of FSS, when the time spent immobile was recorded during the last 4 min. The FST of control and CMI (1 and 10 mg/kg) *per se* groups were exposed only to the last trail of FSS to perform the FST; 30 min after the last FSS session, all experimental groups were subjected to the open field and tail suspension test, in this sequence of tests, to confirm the stress-induced depressive-like phenotype.

Immediately after the last behavioral test, the mice were anesthetized with isoflurane and samples of blood, prefrontal cortices, and hippocampi were collected and stored at −80°C for biochemistry analyses. The dose of 10 mg/kg was chosen to be investigated in the biochemistry analyses based on the results obtained in the behavioral tests.

For protocol 1, the mice were divided into seven groups: I—vehicle (canola oil and saline); II—1 mg/kg CMI; III—10 mg/kg CMI; IV—repeated FSS + vehicle (canola oil and saline); V—1 mg/kg CMI + repeated FSS; VI—10 mg/kg CMI + repeated FSS; VII—10 mg/kg fluoxetine + repeated FSS. All CMI groups also received saline.

#### Protocol 2

In the second protocol was investigated whether long-term stress is induced by repeated FSS and the chronic administration of CMI reverses the effect of repeated FSS. Animals were randomly divided into five experimental groups. In this protocol, the stressed mice performed the repeated FSS, as mentioned earlier, and CMI or fluoxetine at the dose of 1 mg/kg (*n* = 7 animals/group) were administered to mice once a day during 20 days after the repeated FSS. The first administration of CMI or fluoxetine was 30 min after the last section in the FSS. The control and CMI (1 and mg/kg) *per se* groups were only exposed to the environment. The control group and repeated FSS group received two vehicles (saline and canola oil).

Twenty-four hours after the last administration of vehicles, CMI or fluoxetine, non-stressed and stressed mice performed in the following order the behavioral tests: open field test (OFT), tail suspension test (TST) and forced swimming test (FST).

Immediately after the last behavioral test, the mice were anesthetized with isoflurane and samples of blood, prefrontal cortices, and hippocampi were collected and stored at −80°C for biochemistry analyses.

For protocol 2, the mice were divided into five groups: I—vehicle (canola oil and saline); II—10 mg/kg CMI; III—repeated FSS + vehicle (canola oil and saline); IV—10 mg/kg CMI + repeated FSS; V—10 mg/kg fluoxetine + repeated FSS. All CMI groups also received saline.

### Behavioral Tests

#### Open Field Test

The OFT was carried out to assess the possible effect of the treatments on the locomotor and exploratory activities (Walsh and Cummins, 1976). Mice were placed individually in the center of a box (30 × 30 × 15 cm) divided into nine quadrants of equal areas, and observed for 5 min to report their locomotor (scored by the number of segments crossed with the four paws) and exploratory activities (expressed by the number of time the mice stood on rear limbs).

#### Forced Swimming Test

The FST was conducted as described by Porsolt et al. (1977). Briefly, mice were individually forced to swim in an open cylindrical container (12 cm in diameter and 30 cm in height), containing 20 cm of water at 25 ± 1°C. The total duration of the test was 6 min, but the time of immobility was measured during the last 4-min period. Parameters of floating behavior were defined as immobility with only occasional slight movements required for keeping the body balanced and the nose above water.

#### Tail Suspension Test

The total duration of immobility in the TST (6 min) was measured according to the method described by Steru et al. (1985). Mice were acoustically and visually isolated and were suspended 50 cm above the floor by adhesive tape placed approximately 1 cm from the tip of their tail. During the last 4 min, the immobility duration was observed. Immobility was considered when absence of the escape attempt behavior.

### *Ex vivo* Assays

#### Plasma Corticosterone Assay

Blood was centrifuged at 4,000 *g* for 10 min, and serum was separated for the determination of corticosterone levels. Determination of plasma corticosterone levels was performed according to Zenker and Bernstein (1958). Aliquots of plasma were incubated with chloroform and centrifuged (5 min at 2,500 rpm) followed by the addition of 0.1 M NaOH and another round of centrifugation. After the addition of the fluorescence reagent (H_2_SO_4_ and ethanol 50%), samples were centrifuged (5 min at 2,500 rpm) and incubated at room temperature for 2 h. Fluorescence intensity emission, corresponding to plasma corticosterone levels, was recorded at 540 nm of emission and 247 nm of excitation, and corticosterone levels were expressed as nanograms per milliliter.

#### Tissue Preparation

The samples of prefrontal cortices and hippocampi were homogenized in 50 mM Tris-HCl pH 7.4 (1:10, *w*/*v*). The homogenate was centrifuged at 2,400× *g* for 10 min at 4°C, and a low-speed supernatant fraction (S1) was used for the following determinations: the levels of RS formation, thiobarbituric acid reactive species (TBARS), superoxide dismutase (SOD), and catalase (CAT) activities.

#### Determination of the Reactive Species

Quantification of RS levels in the prefrontal cortices and hippocampi of mice were performed according to Loetchutinat et al. (2005). Briefly, aliquots of the homogenate supernatant were incubated with 1 mM dichloro-dihydrofluorescein diacetate (DCHF-DA) and 10 mM Tris–HCl pH 7.4. The oxidation of DCFH-DA to fluorescent dichlorofluorescein (DCF) is measured for the detection of intracellular RS. The DCF fluorescence intensity emission was recorded at 520 and 480 nm of excitation and RS levels were expressed as RS levels per milligram of protein.

#### Thiobarbituric Acid Reactive Species Assay

Lipid peroxidation in the prefrontal cortices and hippocampi was measured by the formation of TBARS during an acid-heating reaction (Ohkawa et al., 1979). An aliquot of the homogenate supernatant was incubated with 8.1% SDS, 0.8% TBA, and acetic acid/HCl (pH 3.4) at 95°C during 1 h. Malondialdehyde was used as a biomarker of lipid peroxidation. Absorbance was measured at 532 nm, and the results were expressed as TBARS levels per milligram of protein.

#### Catalase Activity

The CAT activity was assessed spectrophotometrically by the method described by Aebi (1984), which involves monitoring the disappearance of H_2_O_2_ in the presence of S1 at 240 nm. Enzymatic activity was expressed in units per milligram of protein (1 U decomposes 1 μmol of H_2_O_2_ per minute at pH 7 at 25°C).

#### Superoxide Dismutase Activity

The measurement of SOD activity is based on the capacity of SOD to inhibit autoxidation of adrenaline to adrenochrome (Misra and Fridovich, 1972). The color reaction was detected spectrophotometrically at 480 nm and the enzymatic activity was expressed as units per milligram of protein.

#### Protein Determination

Protein concentration was determined by the method previously described by Bradford (1976) using bovine serum albumin (1 mg/ml) as a standard.

### Statistical Analysis

The data of CMI and repeated FSS were analyzed by two-way ANOVA (CMI × repeated FSS) followed by the Newman–Keuls test. Main effects are presented only when the first-order interaction was non-significant. Comparisons between fluoxetine and repeated FSS/vehicle groups were performed by the one-way ANOVA followed by the Newman–Keuls test. Pearson’s correlation analysis was performed to investigate any possible relationship between the immobility time in the TST, FST, and CMI treatments and biochemistry data. Descriptive statistics data were expressed as the mean(s) ± SEM. Probability values less than 0.05 (*p* < 0.05) were considered as statically significant.

## Results

### Experimental Protocol 1

#### Acute CMI Treatment Protected the Depressive-Like Behavior Induced by Repeated FSS

**Figure 2:**
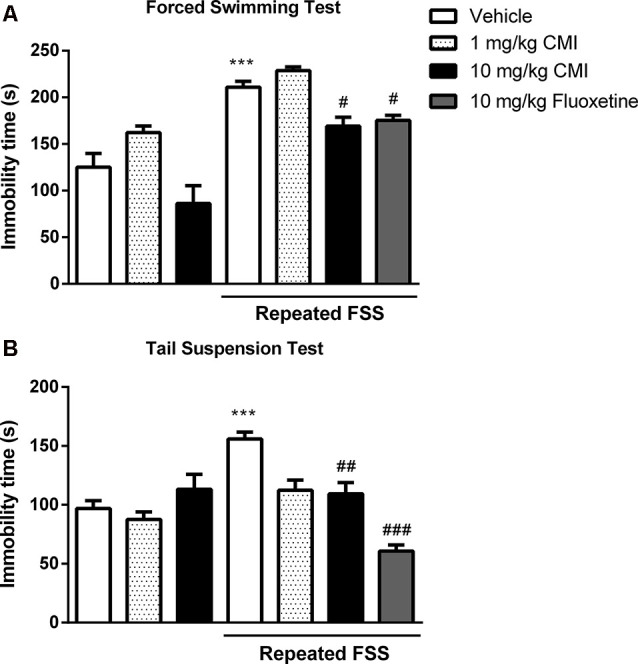
Acute CMI treatment protects against depression-like behavior induced by repeated forced swim stress (FSS). Effects of CMI (1 and 10 mg/kg, i.g.) and fluoxetine (10 mg/kg, i.g.) on depression-like behavior of mice in the forced swimming test (FST; **A**) and tail suspension test (TST; **B**). Values are expressed as mean ± SEM of seven animals/group, especially fluoxetine group which has six animals/group. ***p < 0.001 when compared with the control group. ^#^*p* < 0.05, ^##^*p* < 0.01, and ^###^*p* < 0.001 when compared with the repeated FSS group (one-way or two-way ANOVA followed by the Newman–Keuls test). CMI, 3-((4-chlorophenyl)selanyl)-1-methyl-1H-indole.

Figure 2 shows the immobility time of mouse in the FST (Figure 2A) and TST (Figure 2B). The two-way ANOVA of immobility time in the FST revealed a significant main effect of CMI (*F*_(2,36)_ = 17.88, *p* < 0.001) and repeated FSS (*F*_(1,36)_ = 70.84, *p* < 0.001). The two-way ANOVA of immobility time in the TST revealed a significant CMI × repeated FSS interaction (*F*_(2,36)_ = 7.95, *p* < 0.001).

Repeated FSS induced mouse depressive-like behavior showed by an increase in immobility time in FST and TST tests when compared with that of the control group, and CMI at a dose of 10 mg/kg, but not at the dose of 1 mg/kg, was effective against this increase.

The one-way ANOVA demonstrated a significant effect of repeated FSS and fluoxetine in the FST (*F*_(2,17)_ = 19.53, *p* < 0.001) and in the TST (*F*_(2,17)_ = 51.36, *p* < 0.0001). The fluoxetine in stressed mice decreased the immobility time in the FST and TST.

#### Acute CMI Effects on Spontaneous Locomotor Activity

Table 1 shows the effect of CMI and fluoxetine treatment and repeated FFS on the mouse spontaneous locomotor activity. Neither repeated FSS nor CMI and fluoxetine affected the numbers of crossings and rearings.

**Table 1:** 3-((4-chlorophenyl)selanyl)-1-methyl-1H-indole (CMI) effects on parameters of spontaneous locomotor activity in mice of protocol 1.

Acute treatment	Crossings	Rearings
Control	64 ± 9	20 ± 6
CMI 1 mg/kg	48 ± 9	11 ± 3
CMI 10 mg/kg	77 ± 8	19 ± 3
Repeated FSS	79 ± 8	16 ± 2
Repeated FSS + CMI 1 mg/kg	57 ± 8	20 ± 5
Repeated FSS + CMI 10 mg/kg	66 ± 12	15 ± 3
Repeated FSS + Fluoxetine 10 mg/kg	77 ± 6	17 ± 2

*Values are expressed as means ± SEM (n = 7); differences were not significant. Crossings and rearings were expressed as number*.

#### Acute CMI Treatment Alters Corticosterone Levels

**Figure 3:**
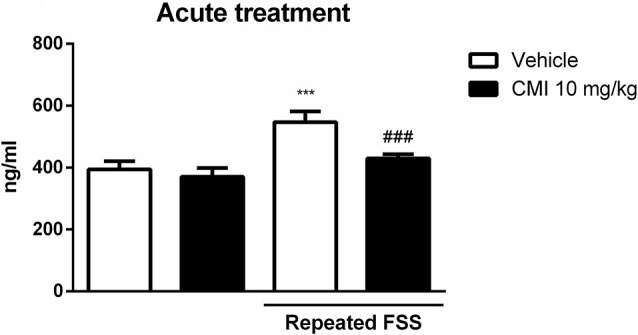
Acute CMI treatment regulates the corticosterone levels altered by repeated FSS in the plasma of mice. Effects of acute CMI treatment (10 mg/kg, i.g.) on the plasma corticosterone levels of mice subjected to repeated FSS. Values are expressed as mean ± SEM of six animals/group. ***p < 0.001 when compared with the vehicle group and ^###^*p* < 0.001 when compared with the repeated FSS control group (two-way ANOVA followed by the Newman–Keuls test). CMI, 3-((4-chlorophenyl)selanyl)-1-methyl-1H-indole.

Figure 3 shows the corticosterone levels in the plasma of mice submitted to acute CMI treatment. The two-way ANOVA of corticosterone levels revealed a significant main effect of repeated FSS (*F*_(1,20)_ = 15.18, *p* < 0.001) and CMI treatment (*F*_(1,20)_ = 6.79, *p* = 0.016).

The *post hoc* test demonstrated that the corticosterone levels in the plasma were increased in mice subjected to repeated FSS when compared with that of the control group, and the acute CMI treatment was effective in protecting this alteration.

#### Acute CMI Treatment Modulated the Oxidative Stress in Prefrontal Cortices and Hippocampi

**Figure 4:**
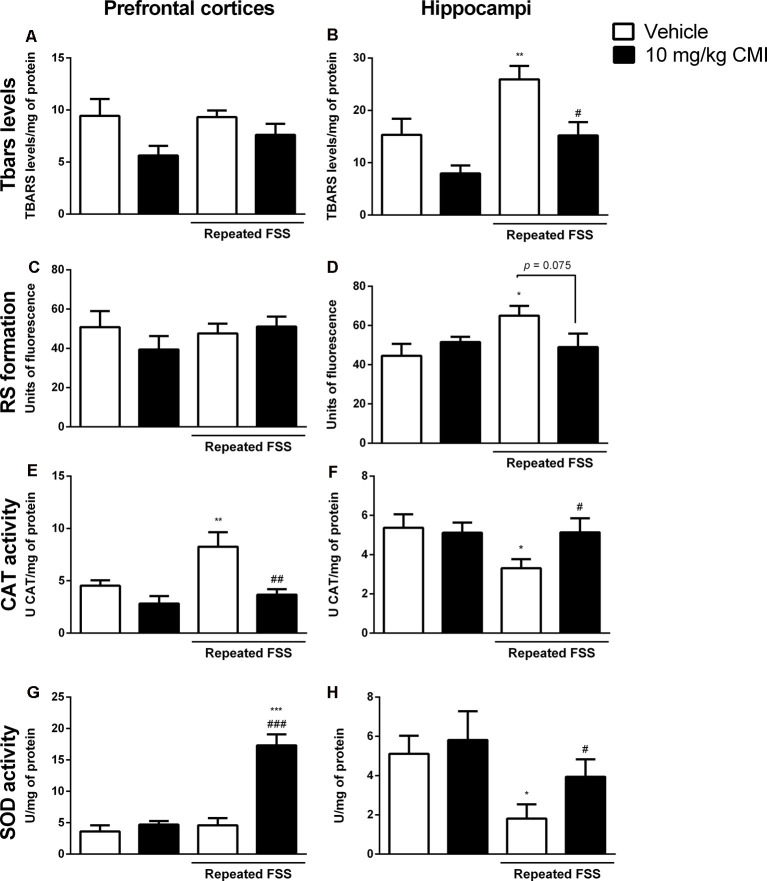
Acute CMI treatment regulates the oxidative stress altered by repeated FSS in the prefrontal cortices and hippocampi of mice. Effects of acute CMI treatment (10 mg/kg, i.g.) on the TBARS levels in the prefrontal cortices **(A)** and hippocampi **(B)**, RS in the prefrontal cortices **(C)** and hippocampi **(D)**, catalase (CAT) activity in the prefrontal cortices **(E)** and hippocampi **(F)**, and SOD activity in the prefrontal cortices **(G)** and hippocampi **(H)** of mice subjected to repeated FSS. Values are expressed as mean ± SEM of 6–7 animals/group. *p < 0.05, **p < 0.01, and ***p < 0.001 when compared with the vehicle group; ^#^*p* < 0.05, ^##^*p* < 0.01, and ^###^*p* < 0.001 when compared with the repeated FSS control group (two-way ANOVA followed by the Newman–Keuls test). CMI, 3-((4-chlorophenyl)selanyl)-1-methyl-1H-indole.

Figures 4A–H shows the involvement of oxidative stress in the antidepressive effect of acute CMI in the repeated FSS model in mice.

The two-way ANOVA of TBARS levels revealed a significant main effect of CMI (*F*_(1,24)_ = 13.18, *p* = 0.001) and of repeated FSS (*F*_(1,24)_ = 12.74, *p* = 0.001) in the hippocampi (Figure 4B). In the prefrontal cortices, neither repeated FSS nor CMI treatment altered the TBARS levels (Figure 4A).

The *post hoc* analysis demonstrated that repeated FSS induced an increase in TBARS levels of the hippocampi when compared with that of the control group, which was protected by CMI treatment.

The two-way ANOVA of RS formation demonstrated a significant interaction of repeated FSS × CMI (*F*_(1,24)_ = 6.45, *p* = 0.017) in the hippocampi (Figure 4D). In the prefrontal cortices, neither repeated FSS nor CMI treatment altered the RS formation (Figure 4C).

Repeated exposure to FSS increased the RS formation in the hippocampi when compared with that of the control group, and this alteration was protected by CMI treatment.

The two-way ANOVA of CAT activity in the prefrontal cortices showed a significant main effect of repeated FSS (*F*_(1,21)_ = 6.12, *p* = 0.021) and CMI (*F*_(1,21)_ = 11.50, *p* = 0.002; Figure 4E), whereas in the hippocampi the two-way ANOVA of CAT activity showed a significant interaction of repeated FSS × CMI (*F*_(1,21)_ = 4.14, *p* = 0.05; Figure 4F).

Repeated FSS increased the CAT activity in the prefrontal cortices when compared with that of the control group, and the CMI treatment protected this alteration. In the hippocampi, repeated FSS decreased the CAT activity and the CMI increased the activity of this enzyme.

The two-way ANOVA of SOD activity in the prefrontal cortices showed a significant interaction repeated FSS × CMI (*F*_(1,23)_ = 23.50, *p* < 0.001; Figure 4G). In the hippocampi, the two-way ANOVA of SOD activity demonstrated a significant main effect of repeated FSS (*F*_(1,20)_ = 9.16, *p* = 0.006; Figure 4H).

Repeated FSS together with CMI treatment increased the SOD activity in the prefrontal cortices when compared with that of the control group and repeated FSS group. In the hippocampi, repeated FSS decreased the SOD activity when compared with that of the control group, and the CMI treatment protected this alteration.

#### Correlations Between Behavioral and Biochemical Effects in the Prefrontal Cortices and Hippocampi of Mice

Considering that the acute administration of CMI reduced the immobility time in the FST and TST and modulated several biochemical endpoints altered by repeated FSS, we analyzed if these effects were correlated using Pearson’s correlation analysis (Table 2). The results demonstrated a significant positive correlation between the immobility time in the FST and corticosterone levels in the plasma, TBARS levels, RS formation in the hippocampi, and CAT activity in the prefrontal cortices. A significant negative correlation was found between the immobility time in the FST and SOD activity in the hippocampi.

**Table 2:** Pearson’s correlation between the immobility time (s) in the tail suspension test (TST), forced swimming test (FST) and biochemical endpoints in the prefrontal cortices and hippocampi of mice submitted to repeated forced swim stress (FSS) and acute CMI treatment.

Immobility time FST				Immobility time TST			
		*r*	*p*			*r*	*p*
Corticosterone levels	Plasma	0.5535	0.0050	Corticosterone levels	Plasma	0.5992	0.0020
TBARS levels	Prefrontal cortices	0.3146	0.1343	TBARS levels	Prefrontal cortices	0.0467	0.8283
	Hippocampi	0.6551	0.0005		Hippocampi	0.5106	0.0108
RS formation	Prefrontal cortices	0.1341	0.5322	RS formation	Prefrontal cortices	0.0202	0.9253
	Hippocampi	0.4425	0.0304		Hippocampi	0.2988	0.1561
CAT activity	Prefrontal cortices	0.5491	0.0055	CAT activity	Prefrontal cortices	0.4774	0.0183
	Hippocampi	−0.1779	0.4056		Hippocampi	−0.2822	0.1816
SOD activity	Prefrontal cortices	0.1835	0.3907	SOD activity	Prefrontal cortices	−0.2134	0.3168
	Hippocampi	−0.6963	0.0002		Hippocampi	−0.4508	0.0270

The results demonstrated a significant positive correlation between the immobility time in the TST and corticosterone levels in the plasma, TBARS levels in the hippocampi, and CAT activity in the prefrontal cortices. A significant negative correlation was found between the immobility time in the TST and SOD activity in the hippocampi.

### Experimental Protocol 2

#### Chronic CMI Effects on Spontaneous Locomotor Activity

Table 3 shows the effect of CMI and fluoxetine treatment and repeated FFS on the mouse spontaneous locomotor activity. Neither repeated FSS nor CMI and fluoxetine affected the numbers of crossings and rearings.

**Table 3:** CMI effects on parameters of spontaneous locomotor activity in mice of protocol 2.

Chronic treatment	Crossings	Rearings
Control	106 ± 5	30 ± 3
CMI 1 mg/kg	105 ± 4	26 ± 2
Repeated FSS	94 ± 3	30 ± 5
Repeated FSS + CMI 1 mg/kg	95 ± 3	27 ± 4
Repeated FSS + Fluoxetine 1 mg/kg	107 ± 4	27 ± 3

*Values are expressed as means ± SEM (n = 7); differences were not significant. Crossings and rearings were expressed as number*.

#### Chronic CMI Treatment Protected the Depressive-Like Behavior Induced by Repeated FSS

**Figure 5:**
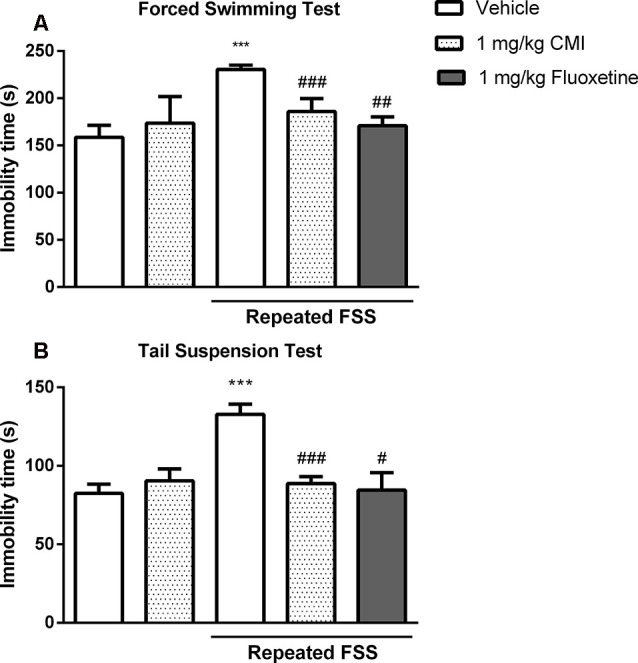
Chronic CMI treatment protects against depression-like behavior induced by repeated FSS. Effects of CMI and fluoxetine (1 mg/kg, i.g.) on depression-like behavior of mice in the FST **(A)** and TST **(B)**. Values are expressed as mean ± SEM of seven animals/group. ***p < 0.001 when compared with the control group. ^#^*p* < 0.05, ^##^*p* < 0.01, and ^###^*p* < 0.001 when compared with the repeated FSS control group (one-way or two-way ANOVA followed by the Newman–Keuls test). CMI, 3-((4-chlorophenyl)selanyl)-1-methyl-1H-indole.

Figure 5 shows the immobility time of mouse in the FST (Figure 5A) and TST (Figure 5B). The two-way ANOVA of immobility time TST revealed a significant CMI × repeated FSS interaction (*F*_(1,24)_ = 17.47, *p* < 0.001) and of immobility time in the FST revealed a significant interaction CMI × repeated FSS (*F*_(1,24)_ = 16.79, *p* < 0.001).

The *post hoc* test demonstrated that 20 days after repeated FSS, the mice demonstrated depressive-like behavior by an increase in immobility time in FST and TST when compared with that of the control group, and chronic administration of CMI at a dose of 1 mg/kg was effective against this increase.

The chronic administration of fluoxetine in stressed mice decreased the immobility time in the FST and TST. The one-way ANOVA demonstrated a significant effect of repeated FSS and fluoxetine in the TST (*F*_(2,18)_ = 8.58, *p* = 0.0024) and in the FST (*F*_(2,18)_ = 17.78, *p* < 0.0001).

#### Chronic CMI Treatment Alter Corticosterone Levels

**Figure 6:**
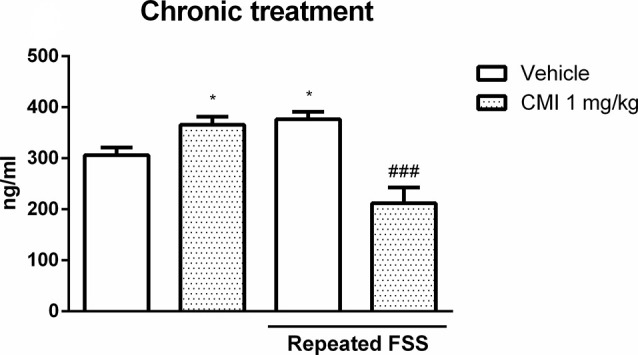
Chronic CMI treatment regulates the corticosterone levels altered by repeated FSS in the plasma of mice. Effects of chronic CMI treatment (1 mg/kg, i.g.) on the plasma corticosterone levels of mice subjected to repeated FSS. Values are expressed as mean ± SEM of six animals/group. *p < 0.05 when compared with the vehicle group and ^###^*p* < 0.001 when compared with the repeated FSS control group (two-way ANOVA followed by the Newman–Keuls test). CMI, 3-((4-chlorophenyl)selanyl)-1-methyl-1H-indole.

Figure 6 shows the corticosterone levels in the plasma of mice submitted to chronic CMI treatment. The two-way ANOVA of corticosterone levels revealed a significant interaction of repeated FSS × chronic CMI (*F*_(1,20)_ = 25.55, *p* < 0.001).

Repeated FSS increased the plasma corticosterone levels when compared with those of the control group, which was restored by chronic CMI treatment. The chronic CMI treatment *per se* increased plasma corticosterone levels.

#### Chronic CMI Treatment Modulated the Oxidative Stress in Prefrontal Cortex and Hippocampus

**Figure 7:**
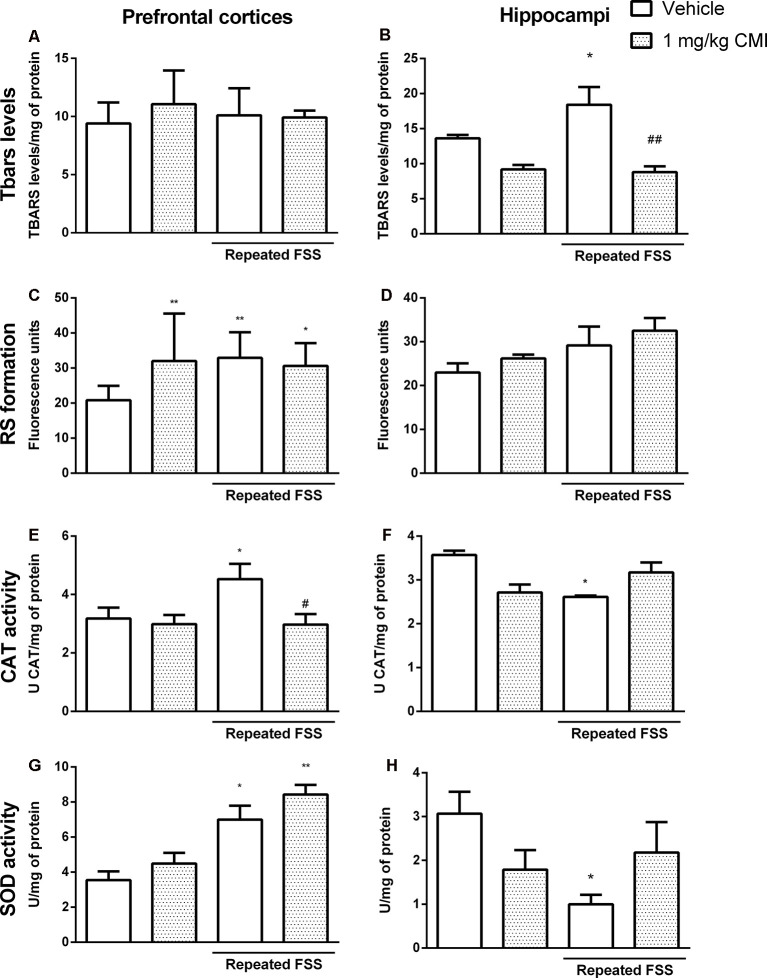
Chronic CMI treatment regulates the oxidative stress altered by repeated FSS in the prefrontal cortices and hippocampi of mice. Effects of chronic CMI treatment (1 mg/kg, i.g.) on the thiobarbituric acid reactive species (TBARS) levels in the prefrontal cortex **(A)** and hippocampi and **(B)**, RS in the prefrontal cortices **(C)** and hippocampi **(D)**, CAT activity in the prefrontal cortices **(E)** and hippocampi **(F)**, and superoxide dismutase (SOD) activity in the prefrontal cortices **(G)** and hippocampi **(H)** of mice subjected to repeated FSS. Values are expressed as mean ± SEM of 6–7 animals/group. *p < 0.05 and **p < 0.01, when compared with the vehicle group; ^#^*p* < 0.05 and ^##^*p* < 0.01 when compared with the repeated FSS control group (two-way ANOVA followed by the Newman–Keuls test). CMI, 3-((4-chlorophenyl)selanyl)-1-methyl-1H-indole.

Figures 7A–H shows the involvement of oxidative stress in the antidepressive effect of chronic CMI in the repeated FSS model in mice.

The two-way ANOVA of TBARS levels in the hippocampi revealed a significant main effect of CMI (*F*_(1,24)_ = 13.48, *p* = 0.003; Figure 7B). Neither repeated exposure to FSS nor chronic CMI treatments were effective in altering the TBARS levels in the prefrontal cortices (Figure 7A).

The repeated FSS increased the TBARS levels in the hippocampi when compared with that of the control group, and the CMI treatment reversed this alteration.

The two-way ANOVA of RS formation demonstrated a significant interaction of CMI × repeated FSS (*F*_(1,24)_ = 12.12, *p* = 0.001) in the prefrontal cortices (Figure 7C) and a significant main effect of repeated FSS (*F*_(1,24)_ = 4.67, *p* < 0.040) in the hippocampi (Figure 7D).

Repeated FSS group and the repeated FSS together with CMI treatment increased the RS levels in the prefrontal cortex of mice when compared with that of the control group. Neither repeated exposure to FSS nor chronic CMI treatment did not alter the RS levels in the hippocampi.

The two-way ANOVA of CAT activity in the prefrontal cortices showed a significant main effect of CMI (*F*_(1,24)_ = 4.73, *p* = 0.039; Figure 7E) and the two-way ANOVA of CAT activity in the hippocampi showed a significant interaction of CMI × repeated FSS (*F*_(1,23)_ = 8.75, *p* = 0.007; Figure 7F).

Repeated FSS increased the CAT activity in the prefrontal cortices when compared with that of the control group, and the CMI treatment protected this alteration. In the hippocampus, repeated FSS decreased CAT activity and the chronic treatment with CMI demonstrated a tendency in increasing CAT activity.

The two-way ANOVA of SOD activity showed a significant main effect of repeated FSS (*F*_(1,20)_ = 16.59, *p* < 0.001) in the prefrontal cortex (Figure 7G). The two-way ANOVA of SOD activity in the hippocampi showed a significant interaction of CMI × repeated FSS (*F*_(1,20)_ = 6.14, *p* < 0.02; Figure 7H).

Repeated FSS alone or together with CMI treatment increased the SOD activity in the prefrontal cortices when compared with that of the control. In the hippocampus, repeated FSS increased SOD activity and the chronic treatment with CMI demonstrated a tendency in increasing the SOD activity.

#### Correlations Between Behavioral and Biochemical Effects in the Prefrontal Cortices and Hippocampi of Mice

Considering that the chronic administration of CMI increased immobility time in the FST and TST and modulated several biochemical endpoints altered by repeated FSS, we analyzed if these effects were correlated using Pearson’s correlation analysis (Table 4). The results demonstrated a significant positive correlation between the immobility time in the FST and RS formation, CAT, and SOD activity in the prefrontal cortices. A significant negative correlation was found between the immobility time in the FST and SOD activity in the hippocampi.

**Table 4:** Pearson’s co ime (s) in the TST, FST and biochemical endpoints in the prefrontal cortices and hippocampi of mice submitted to repeated FSS and chronic CMI treatment.

Immobility time FST				Immobility time TST			
		*r*	*p*			*r*	*p*
Corticosterone levels	Plasma	0.3358	0.1087	Corticosterone levels	Plasma	0.4350	0.0336
TBARS levels	Prefrontal cortices	0.0111	0.9588	TBARS levels	Prefrontal cortices	0.0952	0.6579
	Hippocampi	0.2986	0.1564		Hippocampi	0.5416	0.0063
RS formation	Prefrontal cortices	0.4409	0.0310	RS formation	Prefrontal cortices	0.3120	0.1378
	Hippocampi	0.3351	0.1094		Hippocampi	0.4401	0.0314
CAT activity	Prefrontal cortices	0.5610	0.0043	CAT activity	Prefrontal cortices	0.3700	0.0751
	Hippocampi	−0.3429	0.1010		Hippocampi	−0.4259	0.0380
SOD activity	Prefrontal cortices	0.5163	0.0139	SOD activity	Prefrontal cortices	0.2469	0.2448
	Hippocampi	−0.4945	0.0140		Hippocampi	−0.5159	0.0140

The results demonstrated a significant positive correlation between the immobility time in the TST and corticosterone levels in the plasma, TBARS levels, and RS formation in the hippocampi. A significant negative correlation was found between the immobility time in the TST and CAT and SOD activity in the hippocampi.

## Discussion

The present study demonstrates that: (1) repeated FSS induces long-term stress in mice observed in the FST and TST; and (2) there was an antidepressant-like effect of acute and chronic CMI in a model of depression induced by repeated FSS. The results reported here clearly show that acute and chronic CMI and fluoxetine treatment were effective against depressive-like behavior induced by repeated FSS, without changing their spontaneous locomotor activity. The *ex vivo* analysis findings of this study provide evidence of corticosterone levels and oxidative stress contribution in the CMI action in the depression-like behavior induced by repeated FSS. The role of oxidative stress and the corticosterone levels in the behavioral effects of repeated FSS is supported by the significant correlations between the biochemical endpoints and immobility time in the FST and TST.

To the first aim of the present study, we used the repeated FSS to investigate whether the acute administration of CMI abolishes the stress-induced depressive behavior in mice. The mice exposed to the repeated FSS protocol exhibited greater immobility time, and acute treatment with CMI in the dose of 10 mg/kg was effective in reducing this parameter in the FST and TST, suggesting an antidepressant-like action of CMI in the stress model. In addition, the results showed that only one administration of dose of 1 mg/kg of CMI did not decrease the total immobility time of neither FST nor TST in mice.

In the same way, the antidepressant-like effect elicited by CMI has been already shown by our research group. These studies demonstrated the antidepressant-like effect of CMI in the inflammatory model and acute restraint stress model (Casaril et al., 2017, 2019a). The persistent investigation of new molecules to depressive symptoms is necessary because the depression precipitated by stress occurs in the general population (Yang et al., 2015).

Besides acute stress, it is important to investigate the effects of compounds in the long-lasting effects of stress. So, in this study, we standardized the behavioral change caused 20 days after the exposure to repeated FSS and the effect of CMI and fluoxetine. Collectively, our study revealed the long-term impact of the repeated FSS condition on mouse model, demonstrating that repeated FSS is a significant stressor that induces behavioral and physiologic responses that recapitulate depression-like phenotype.

Our results demonstrate that repeated FSS, a factor similar to real life (repeated and inescapable stress), induces long-term stress. The repeated FSS, a factor similar to real life (repeated and inescapable stress), increased the immobility time in FST and TST (despair behavior), and fluoxetine, a classic antidepressant, was effective in reversing these alterations. This affirmation is supported by: (1) predictive validity, the model’s ability to detect treatments that are clinically useful; (2) face validity, the ability to reproduce the symptoms of human disease in animals; and (3) constructive or etiological validity, which is the requirement for similar causal factors (Willner, 1984; Willner and Mitchell, 2002).

The chronic administration of CMI in the dose of 1 mg/kg improved the responses in the FST and TST behaviors, which indicates the potential antidepressant-like action of this compound in a model induced by long-term stress, a common precipitating factor of emotional disorders in human beings.

Some factors in this behavioral study are especially important and proposed more validity to CMI antidepressant-like effect: (1) the low doses of CMI used in this study; and (2) the CMI had an effect in two moments, when administered concomitantly to the repeated FSS and administered after the stabilization of depression-like behavior.

In the present study, fluoxetine also improved the responses in the FST and TST behaviors in the short-term and long-term stress, but this classic antidepressant was used with the aim of validating the behavioral tests and not with the intent to compare its effect with CMI. The effect of CMI and fluoxetine cannot be compared directly because the pharmacokinetics of both are different. More studies are necessary to elucidate this hypothesis; it can be considered a limitation of the present study.

The mechanisms already described in the literature for the CMI antidepressant-like effect are modulating the oxidative and nitrosative stress, neuroinflammatory process, and serotonergic system; it characterizes this special compound as multi-target. In this study, we prioritize the involvement of oxidative stress in two brain structures, prefrontal cortices and hippocampi, and corticosterone levels in the plasma.

Some changes such as activation of the HPA axis are caused by stress. This neuroendocrine cascade results in the release of the corticotropin-releasing hormone by the hypothalamus and the consequence is releasing pituitary adrenocorticotropin hormone culminating in the secretion of corticosterone in rodents into the circulatory system (Jankord and Herman, 2008).

Considering that the exposure of mice to repeated FSS can generate stress, the current study investigated the corticosterone levels in the plasma of mice in both protocols. Repeated FSS increased the levels of corticosterone in both moments, right after and 20 days after the last section of repeated FSS, and the treatments with CMI decreased this alteration. Thus, it is possible that deleterious consequences on behaviors, caused by repeated FSS, are a consequence of alteration of corticosterone levels and antidepressant effect of CMI can be through the regulation these levels. These findings, together with the significant positive correlation with behavioral tests, show the ability of CMI to modulate hyperactivity of the HPA axis, an effect that can play a role in its antidepressant properties.

The alterations in the HPA axis are caused especially when there is exposure to acute stress (Browne et al., 2014), but some authors demonstrated the alteration in the corticosterone levels caused by long-term stress (Dallman et al., 2004; Bernatova et al., 2018). In addition, HPA axis activation can produce damaging physiological effects and exert a profound impact on brain function (McEwen, 2000; Tsigos and Chrousos, 2002).

Previous studies have shown that the excess of glucocorticoids, especially corticosterone, lead to oxidative stress in the brain of mice (Spiers et al., 2014; Freitas et al., 2015). Oxidative stress is defined as a condition arising from the imbalance between RS and the antioxidant systems, and this imbalance can cause alterations in the brain, a region vulnerable to oxidative stress damage. Furthermore, studies suggest that the increase of oxidative stress in the central nervous system is implicating the mechanism of depressive disorder (Balmus et al., 2016; Salim, 2017).

The results of this study suggest that the repeated FSS, both right after the last section as long term, increased the corticosterone levels and possibly caused the imbalance of oxidative stress. The repeated FSS, when analyzed right after the last section, increased the RS and TBARS levels in the hippocampi, and the acute CMI treatment was effective in reversing the TBARS levels and partially RS formation. In relation to antioxidant enzymes, the repeated FSS altered the CAT activity in the prefrontal cortices and hippocampi and the CMI modulated in both brain structures. The SOD activity was altered by repeated FSS in the hippocampus, and acute CMI treatment reversed this alteration. We suggest that there is an imbalance in the oxidative stress and a compensatory mechanism by CAT and SOD activity. The SOD activity was increased in the prefrontal cortex in stressed mice that received the CMI; therefore, this increase could be a compensatory mechanism. We assume as limitation of this study that more studies are necessary to understand completely the mechanism of CMI in the repeated FSS concerning oxidative stress.

The repeated FSS when analyzing its long-term effect increased the TBARS levels in the hippocampi and increased the RS in the prefrontal cortices; interestingly, the chronic CMI treatment was effective only in reversing the TBARS levels in the hippocampi. The chronic CMI treatment was investigated in relation to antioxidant enzymes, and CMI decreased the catalase activity in the prefrontal cortex, but it did not reduce the SOD activity induced by repeated FSS. In relation to hippocampi, the stress decreases the SOD and CAT activity and the CMI has a tendency to increase both enzymes. Taken together our results, we could suggest that the increase of RS formation by repeated FSS consequently leads to an increase of the catalase and SOD activity, and this increase of catalase activity avoids the lipid peroxidation in the prefrontal cortices. In the hippocampi, the lipid peroxidation in the stressed animals is caused by decreasing of enzymes, SOD and CAT, and the CMI uses both enzymes, together, to decrease the lipid peroxidation.

Other studies demonstrated that CMI treatment regulates the oxidative stress, for example, acute treatment with CMI reversed oxidative stress in the prefrontal cortices and hippocampi of mice subjected to acute restraint stress (Casaril et al., 2019a); CMI pre-treatment prevented RS production induced by lipopolysaccharide in cerebral cortex of mice (Casaril et al., 2017); Birmann et al. (2019) show that CMI also decreased the levels of RS and lipid peroxidation in frontal cortices and hippocampi and plasma levels of corticosterone in mice presenting depressive-like behavior induced by partial sciatic nerve ligation.

Considering the pharmacological actions of CMI in different depression-like models and the fact that the modulation of different neural pathways contributes to its action, we hypothesized that CMI treatment, acute or chronic, could regulate the corticosterone levels and oxidative stress in mice subjected to repeated FSS, contributing to abolish the depressive-like phenotype induced by repeated FSS.

In conclusion, this study demonstrates the effectiveness of CMI in abolishing the depressive-like phenotype induced by short- and long-term stress caused by repeated FSS. In addition, the present study expands our knowledge about the role of corticosterone and oxidative stress in the CMI antidepressant-like effect and suggests that this compound regulates the changes in the prefrontal cortices and hippocampi of mice subjected to repeated FSS.

## Data Availability Statement

The raw data supporting the conclusions of this article will be made available by the authors, without undue reservation.

## Ethics Statement

The animal study was reviewed and approved by Comissão de Ética em Experimentação Animal da Universidade Federal de Pelotas (UFPel).

## Author Contributions

AP: planning, execution, writing of drug treatment, and performed all behavioral and biochemical studies. PB and RP: execution of drug treatment and performed all behavioral and biochemical studies. NP: synthesis of chemical compound and writing this section. EL: planning, correction of the writing, and organic compound synthesis. LS: planning drug treatment, all behavioral and biochemical studies, and correction of the writing.

## Conflict of Interest

The authors declare that the research was conducted in the absence of any commercial or financial relationships that could be construed as a potential conflict of interest.
